# Supplementary figures and images for: Altering mammalian transcription networking with ADAADi: An inhibitor of ATP-dependent chromatin remodeling
Source: PLoS One. 2021 May 17;16(5):e0251354. doi: 10.1371/journal.pone.0251354 (PMC8128233; doi:10.1371/journal.pone.0251354)

S1 Fig

A.

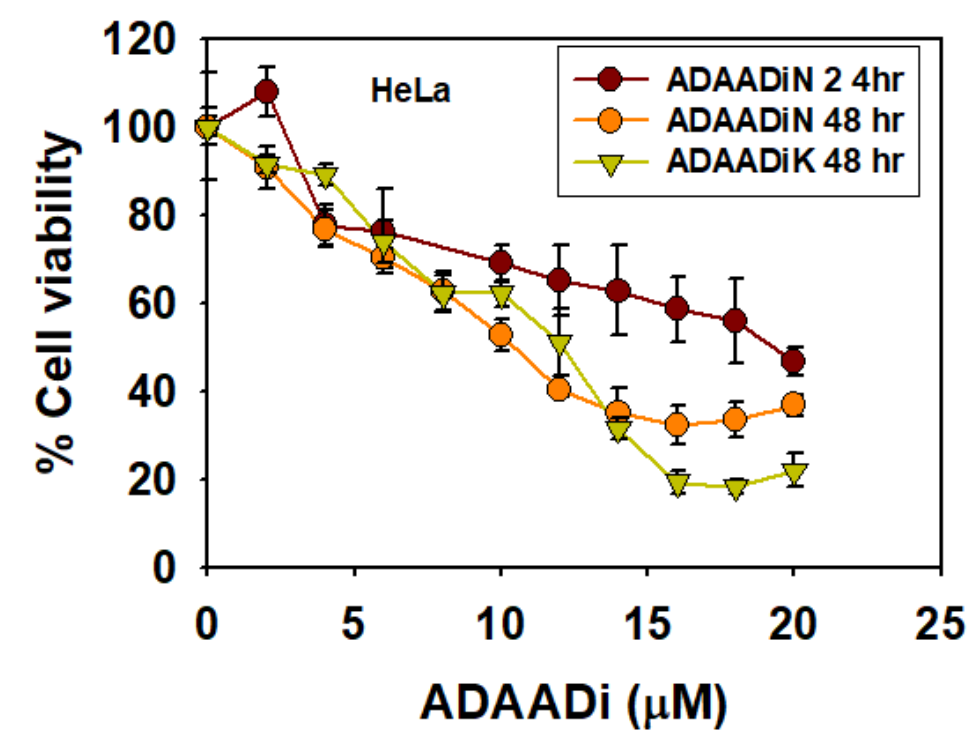

B.

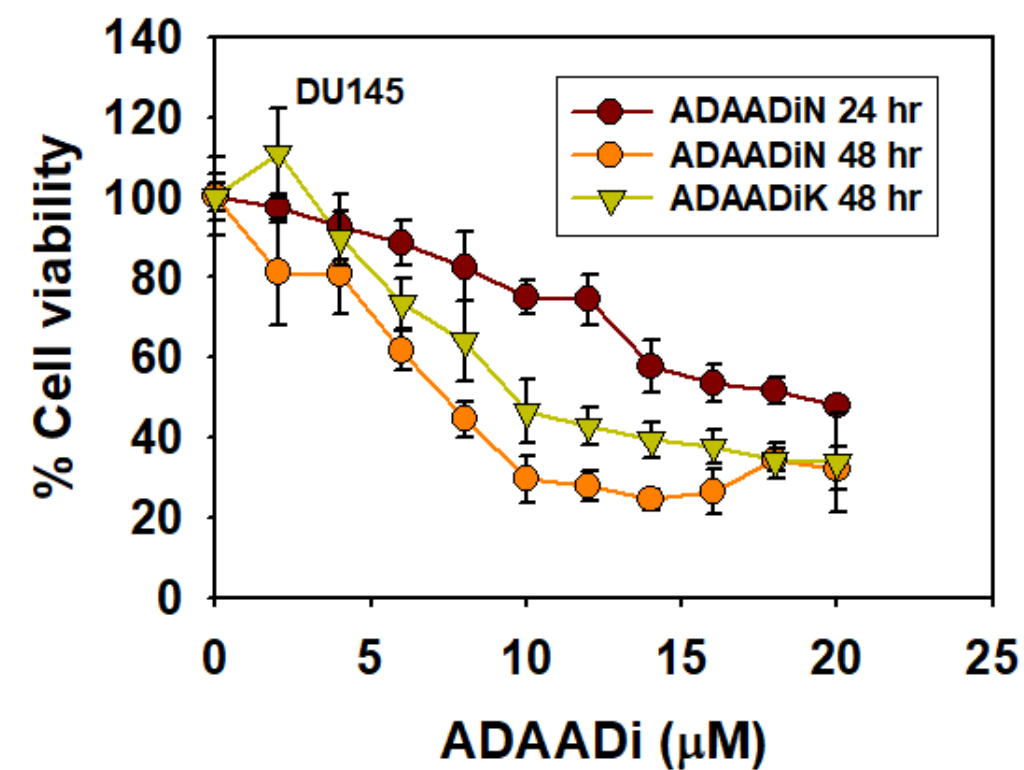

C.

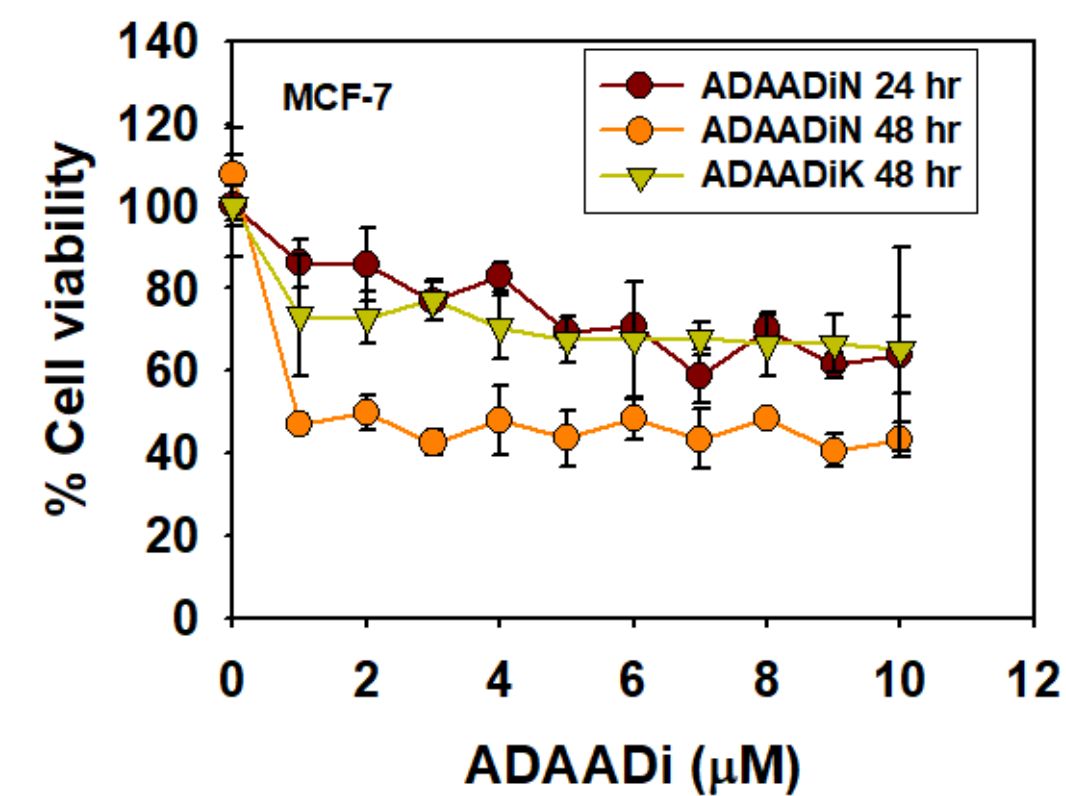

D.

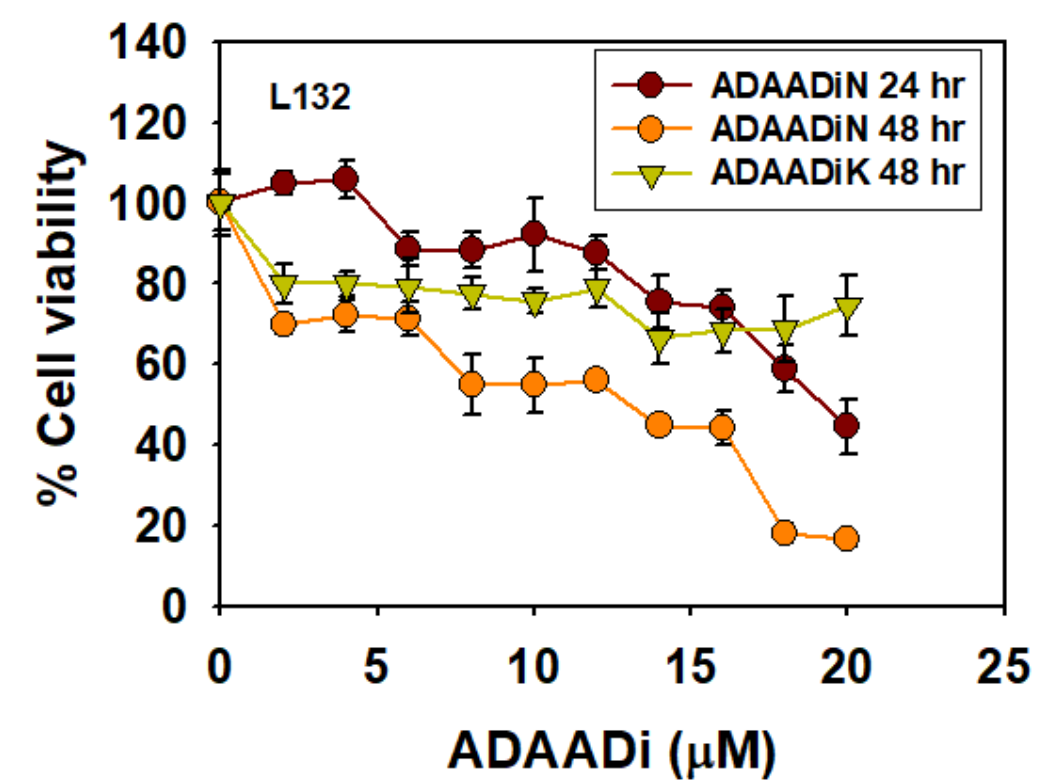

E.

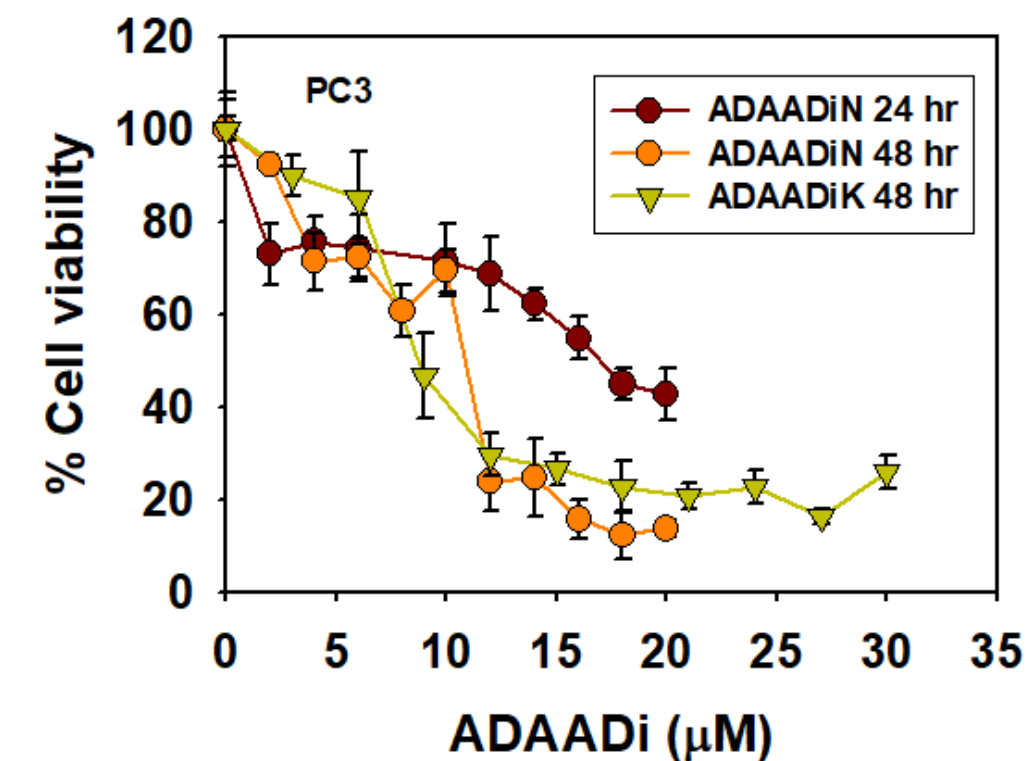

F.

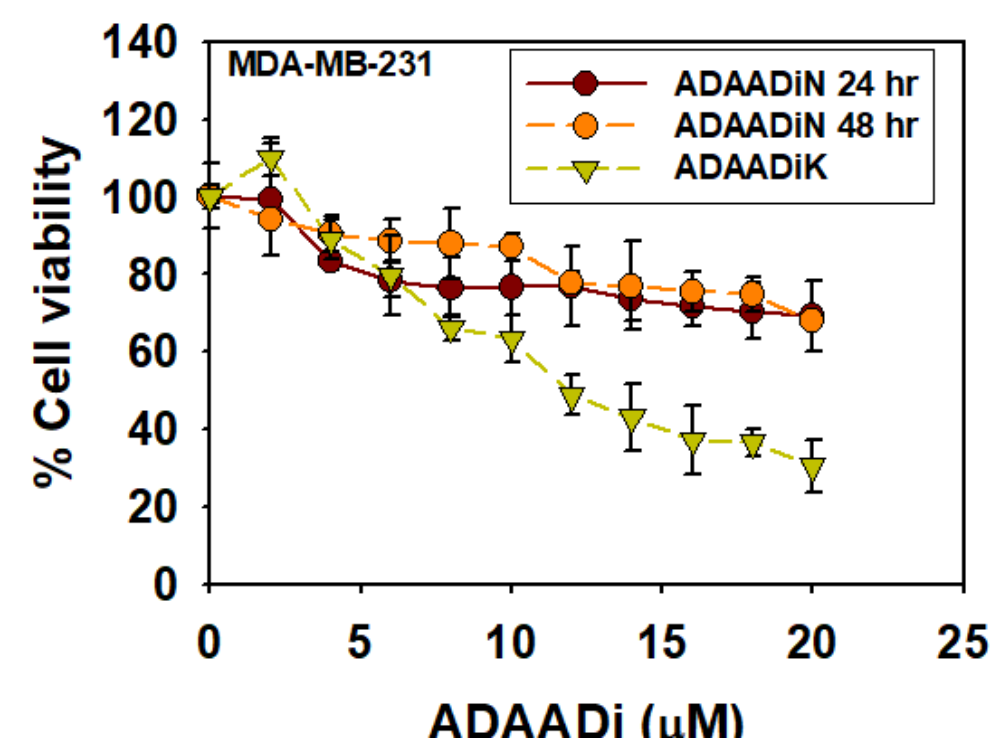

G.

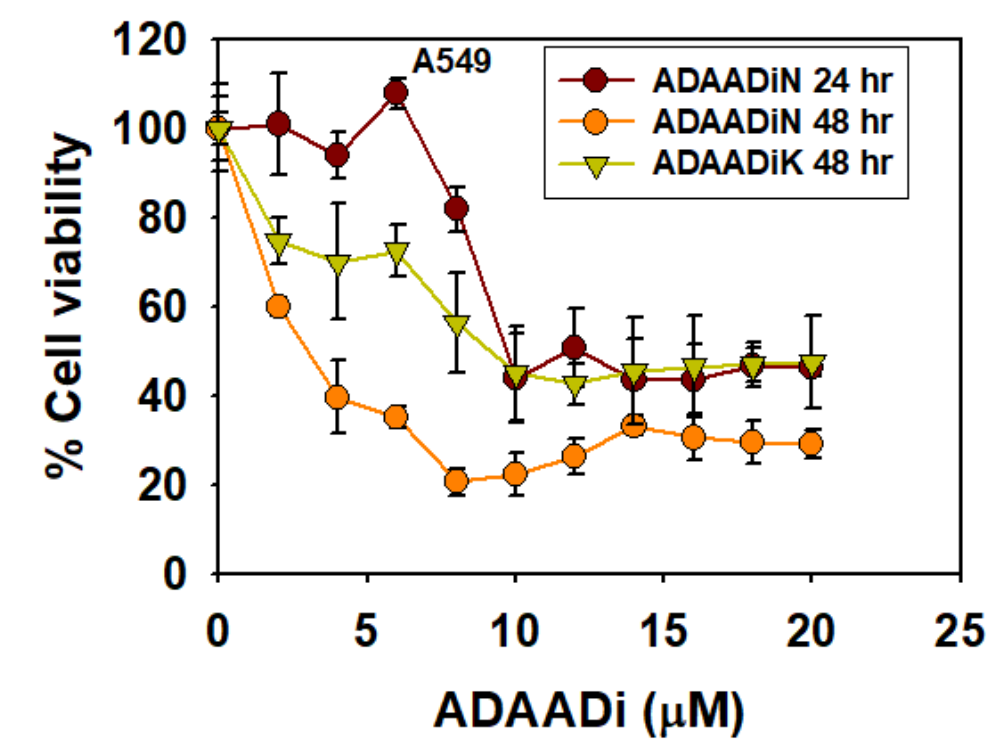

H.

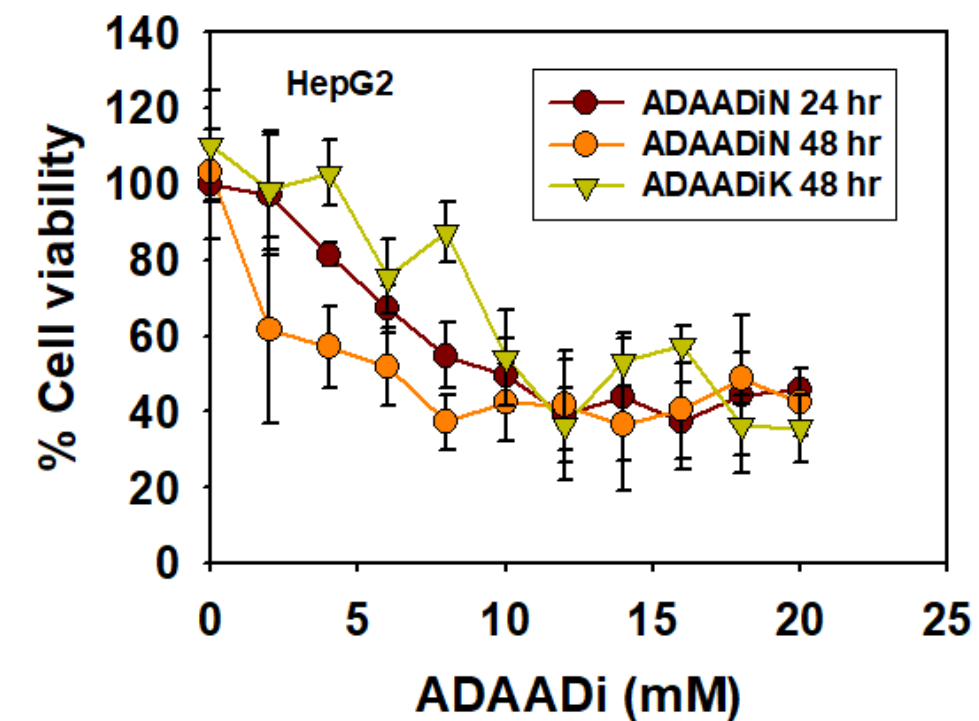

I.

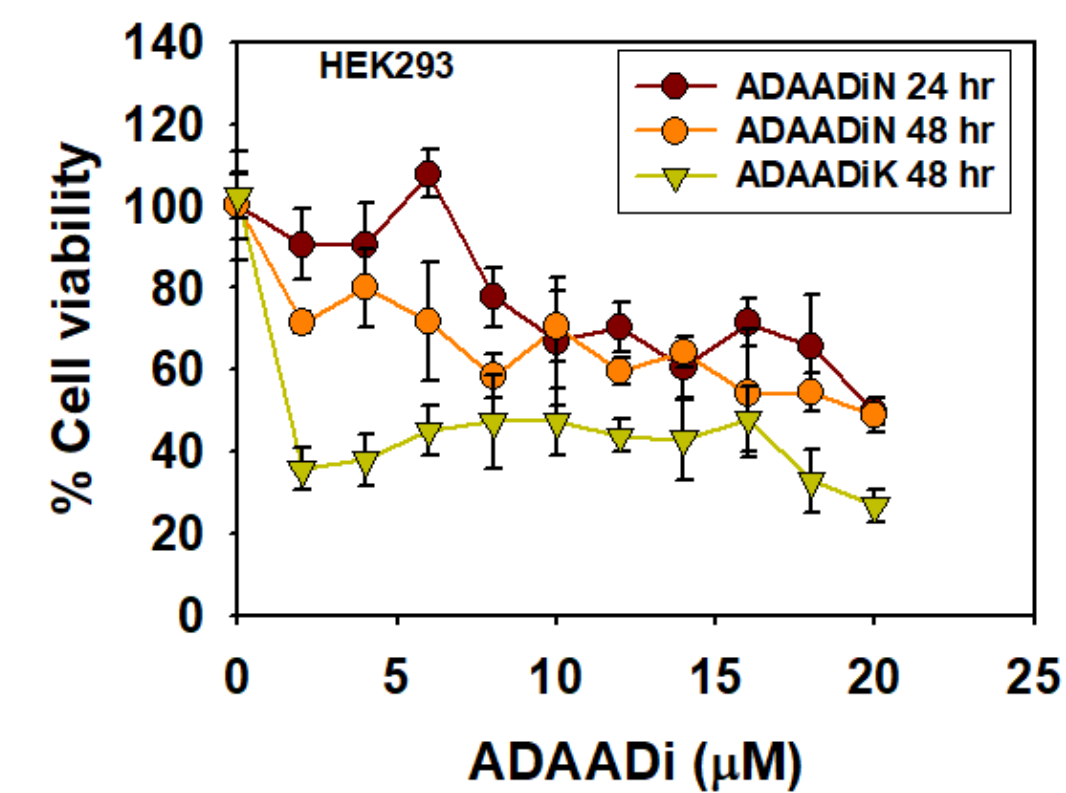

Supplement: S1 Fig — Kill curves showing the effect of ADAADi after treatment. (A). HeLa (B). DU145 (C). MCF-7 (D). L132 (E). PC3 (F). MDA-MB-231 (G). A549 (H) HepG2 (I) HEK293. (PDF) [file pone.0251354.s001.pdf]

S2 Fig

A.

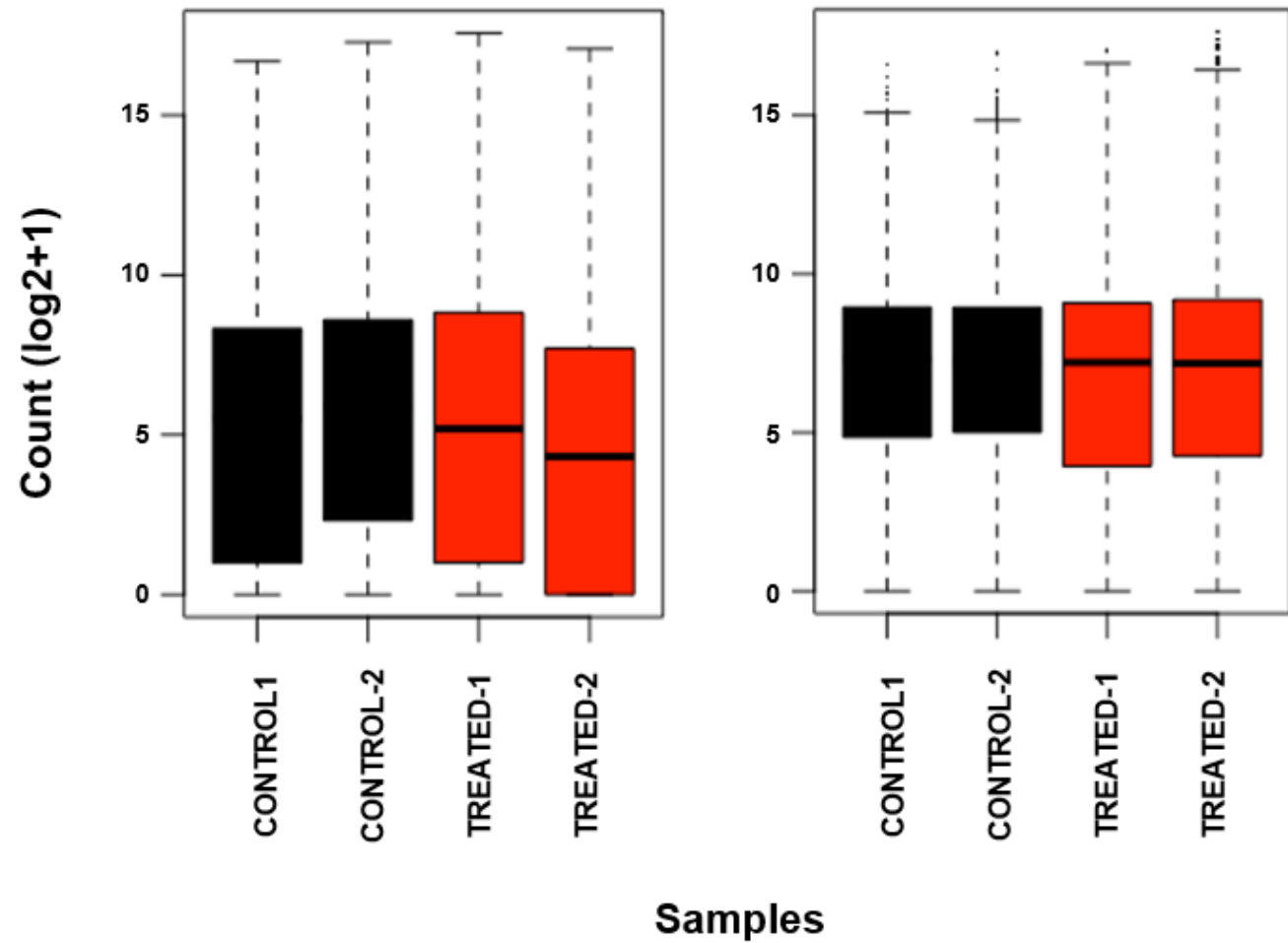

B.

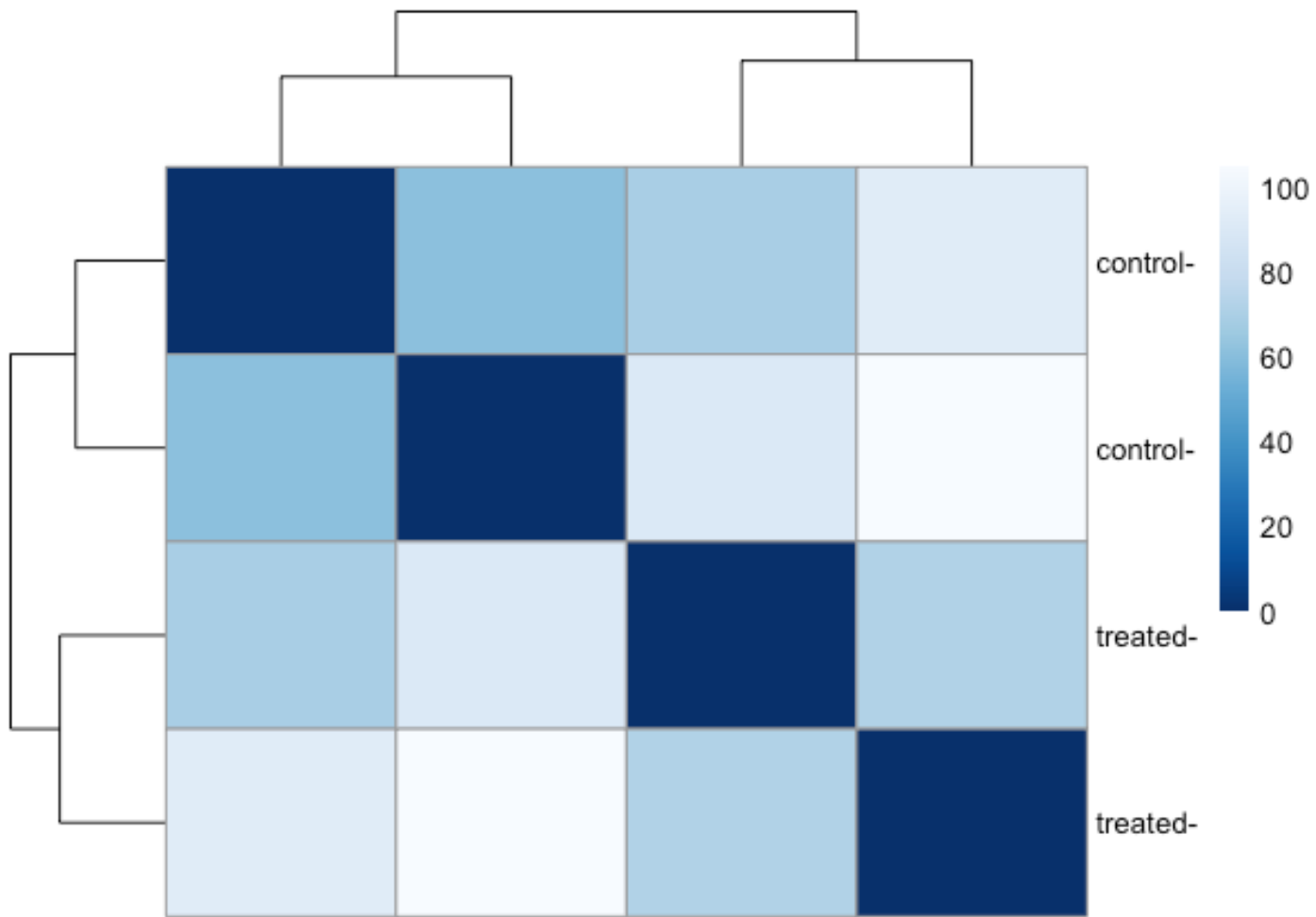

C.

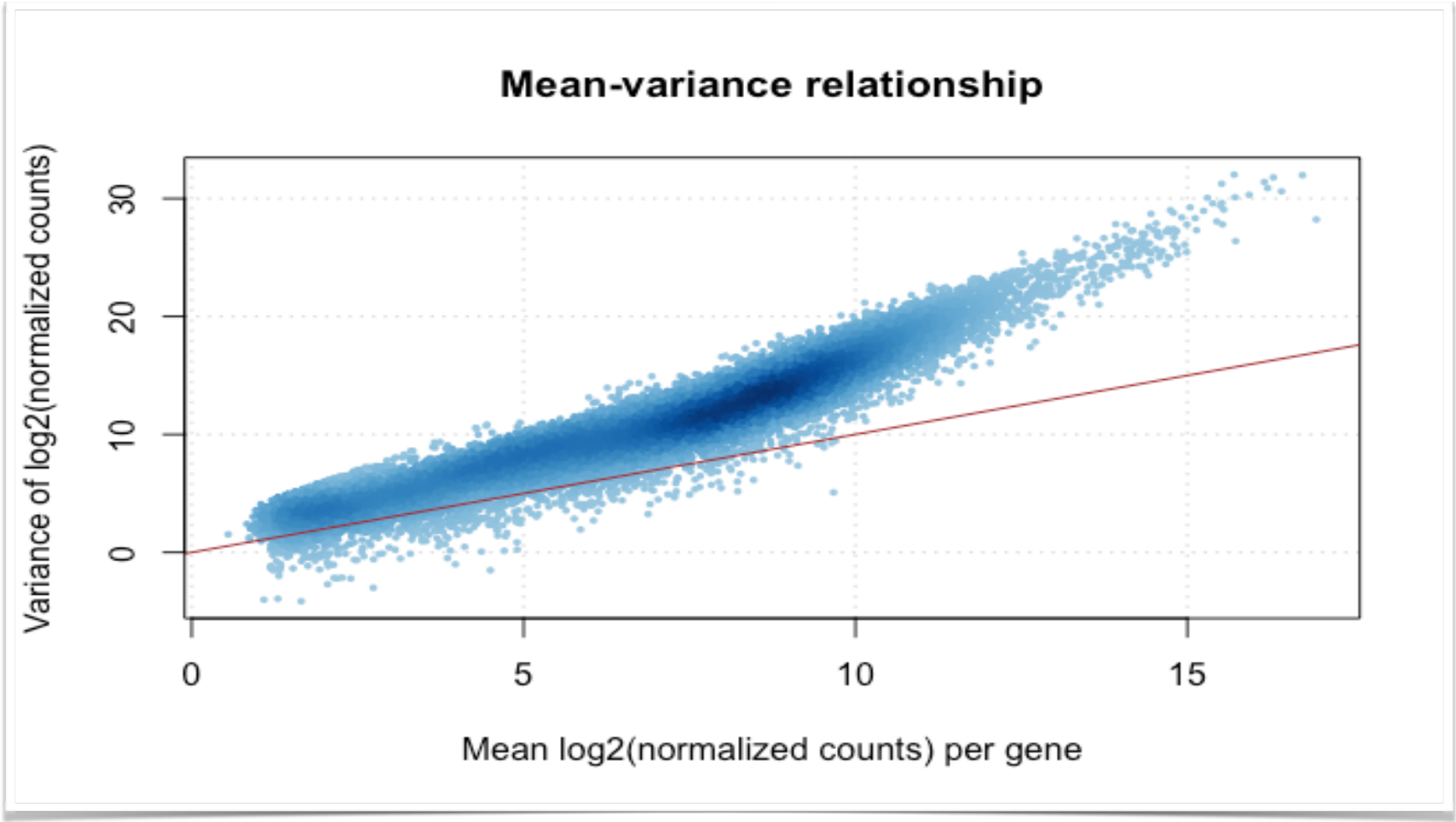

Supplement: S2 Fig — (A). The counts per sample before and after normalization. (B). Mean-variance relationship between the untreated and ADAADi-treated RNA-seq samples. (C). Correlation between untreated and ADAADi-treated RNA-seq samples. (PDF) [file pone.0251354.s002.pdf]

S3 Fig

A.

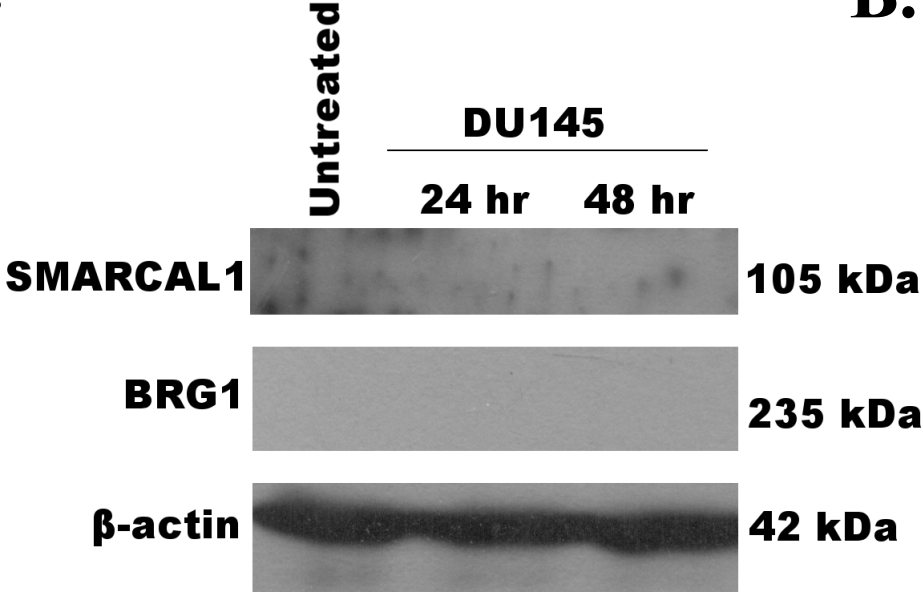

B.

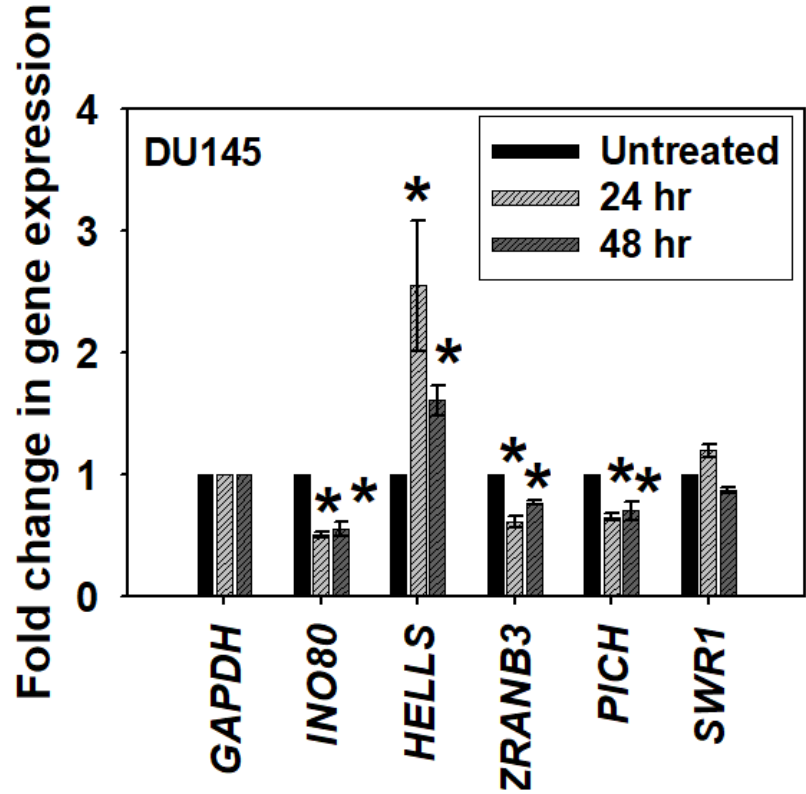

C.

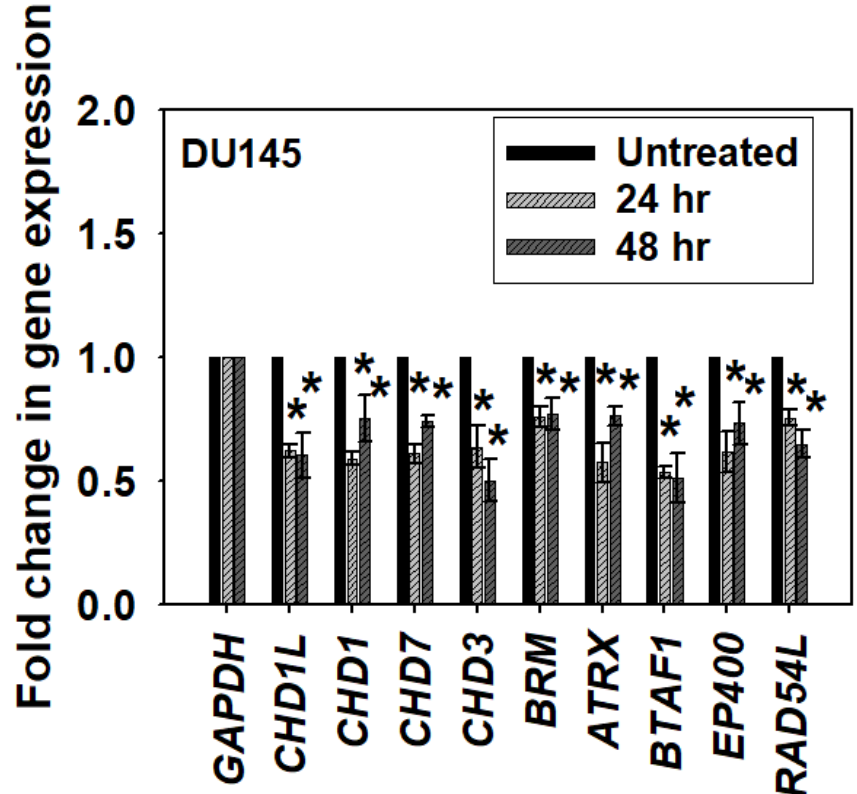

D.

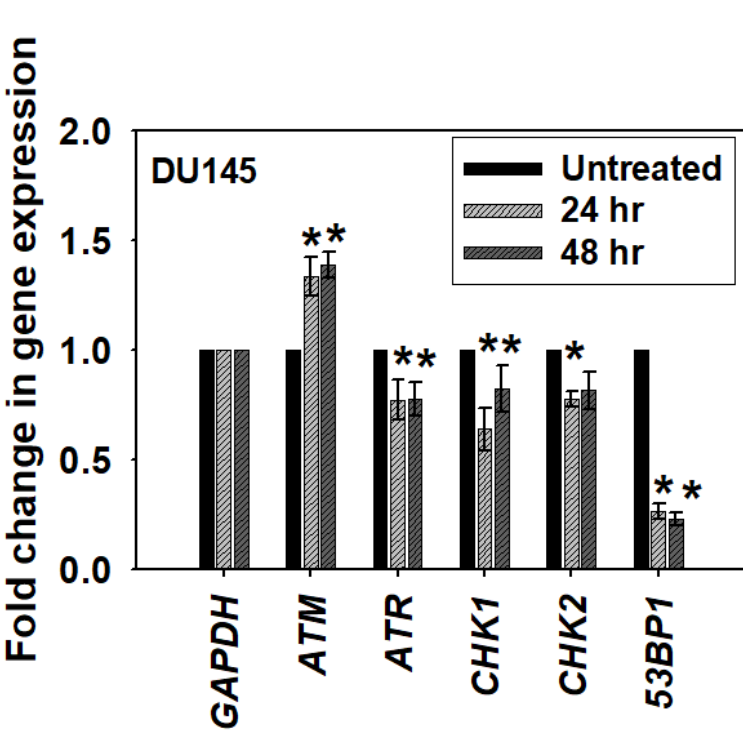

E.

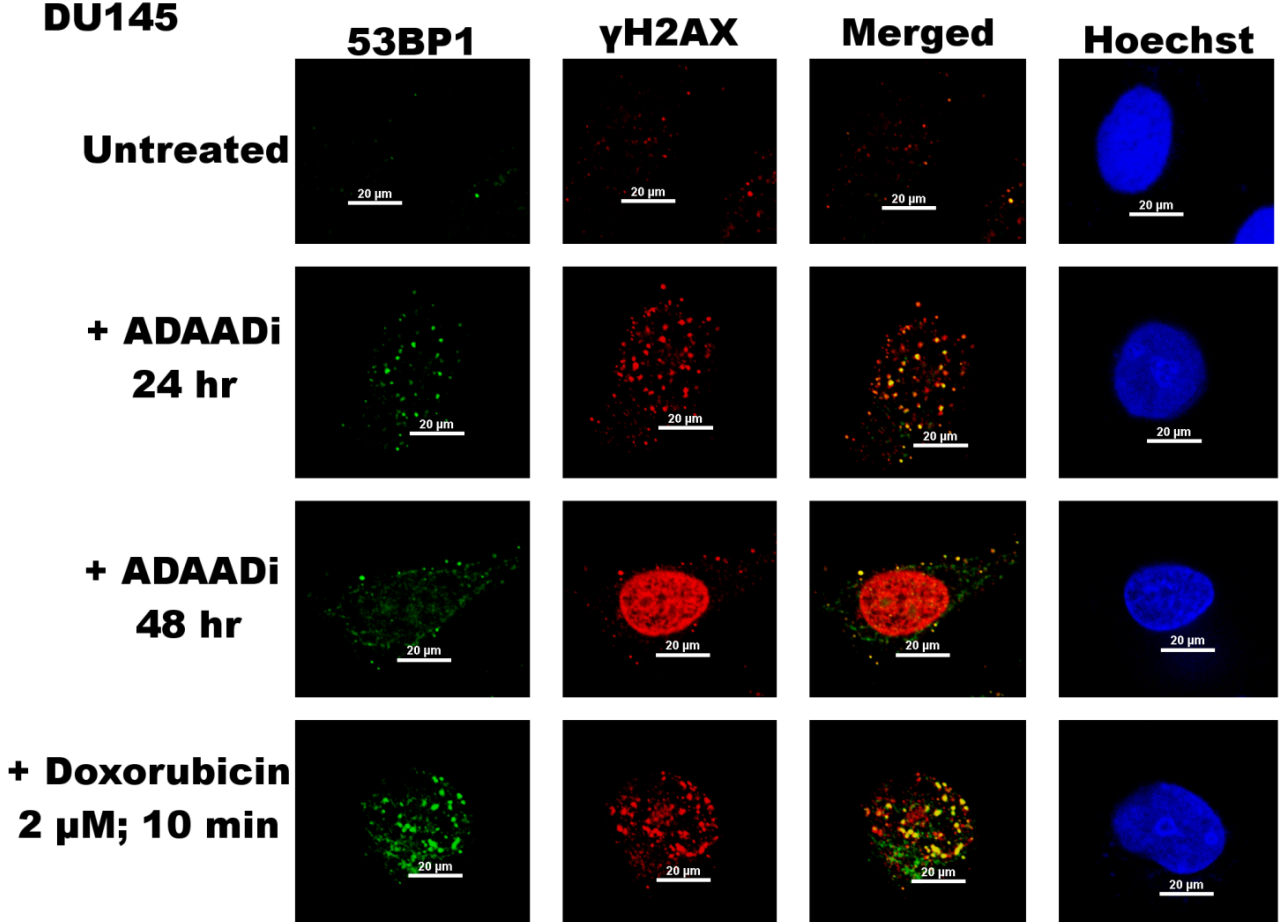

F.

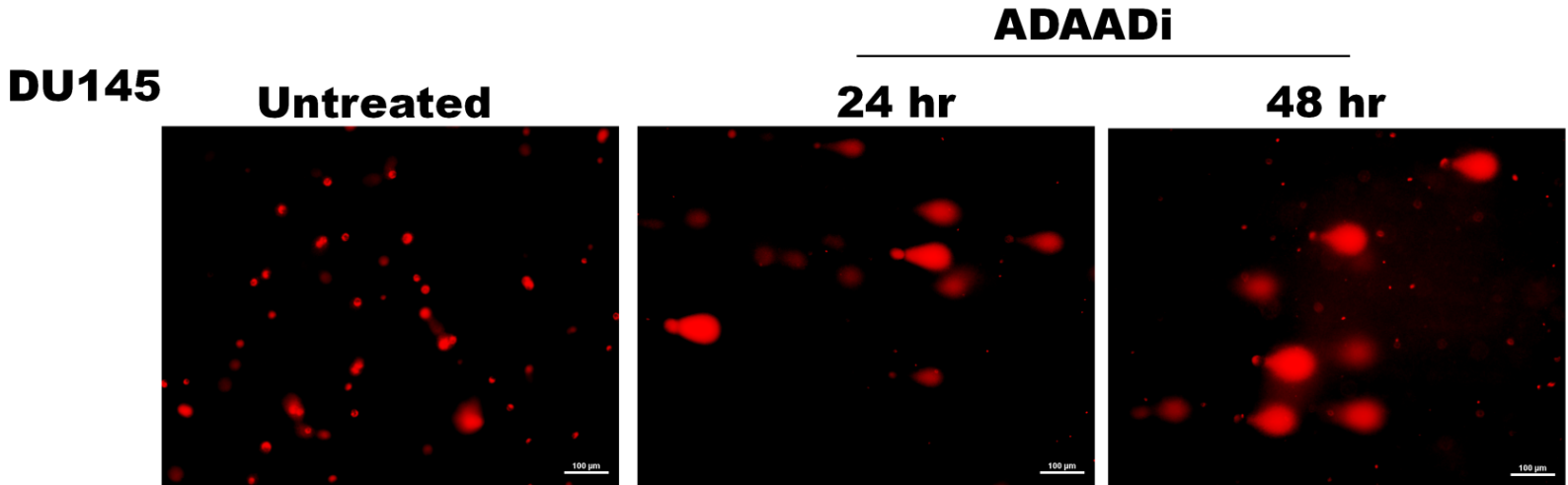

G.

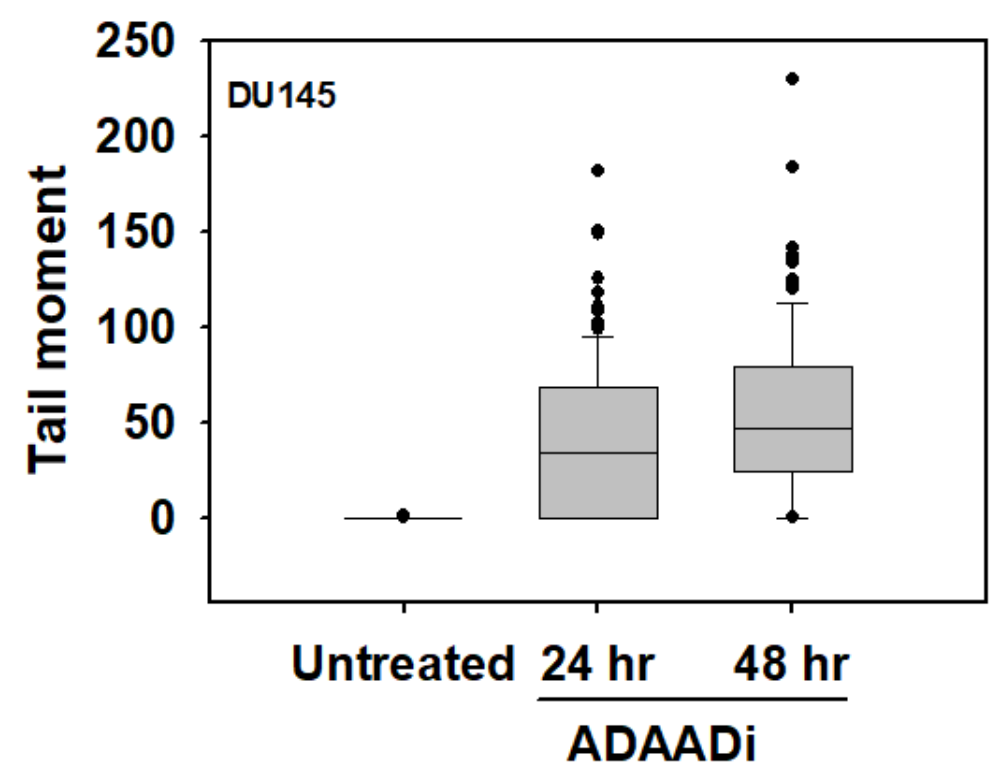

Supplement: S3 Fig — (A). Expression of SMARCAL1 and BRG1 in untreated and 2 μM ADAADi treated cells at 24 and 48 hr. β-actin was used as loading control. (B). The transcript levels of INO80, HELLS, ZRANB3, PICH, and SWR1 was measured using qPCR in DU145 cells after treatment with ADAADi. (C). The transcript levels of CHD1L, CHD1, CHD7, CHD3, BRM, ATRX, BTAF1, EP400, and RAD54L was measured using qPCR in DU145 cells after treatment with ADAADi. (D). The transcript levels of ATM, ATR, Chk1, Chk2, and 53BP1 was estimated using qPCR in DU145 cells after treatment with 2 μM ADAADi. (E). Formation of γH2AX and 53BP1 foci was assessed in DU145 cells after treatment with ADAADi (for 24 and 48 hr) using immunofluorescence. For this experiment, 2 μM doxorubicin treatment for 10 min was used as a positive control. (F). Comet assay in the presence of 100 μM H2O2 treatment for 10 minutes (positive control), untreated, and ADAADi treated (24 and 48 hours) DU145 cells. (G). Quantitation of tail moment in untreated and ADAADi treated DU145 samples. For the qPCR experiments, GAPDH was used as internal control. HeLa cells were treated with 5 μM ADAADi for the time point indicated. The data is presented as average ± s.d of three independent experiments. Statistical analysis was done using Sigma-plot and star indicates significant change at p<0.05. (PDF) [file pone.0251354.s003.pdf]

S4 Fig

A.

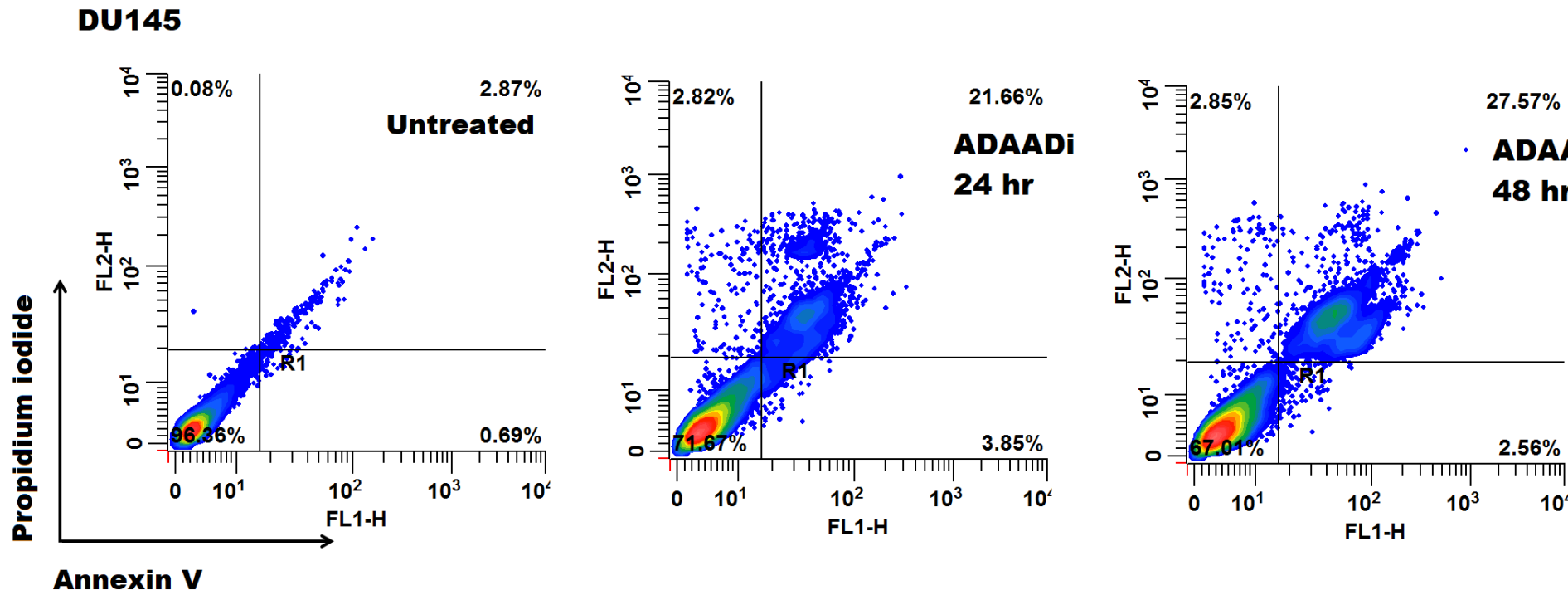

B.

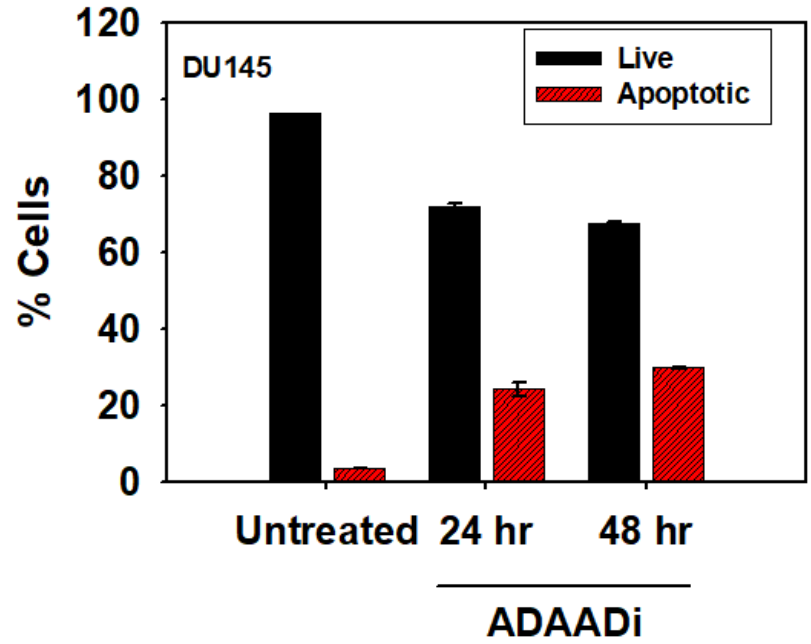

D.

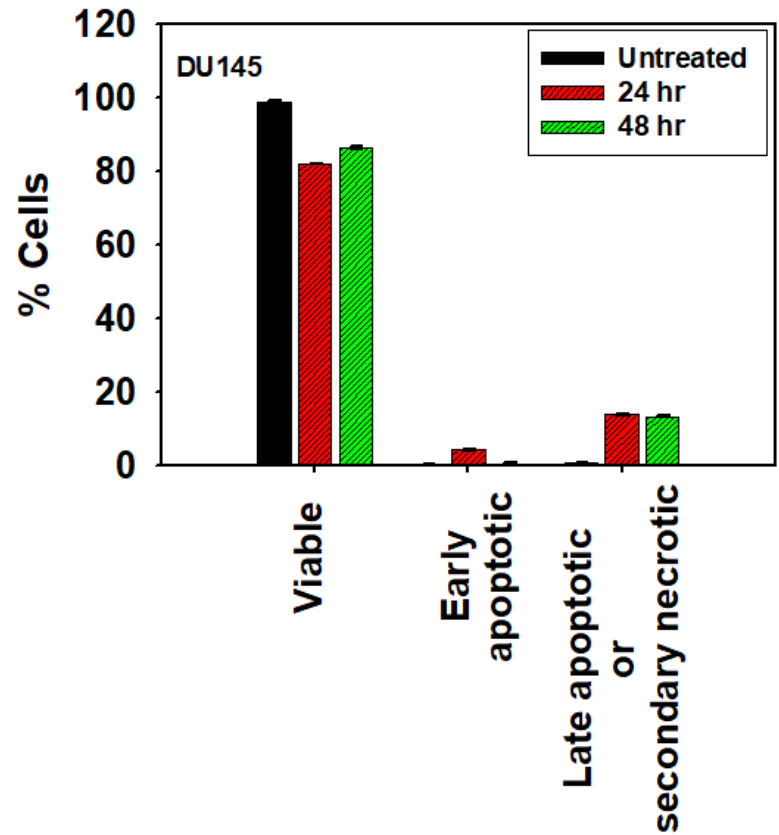

C.

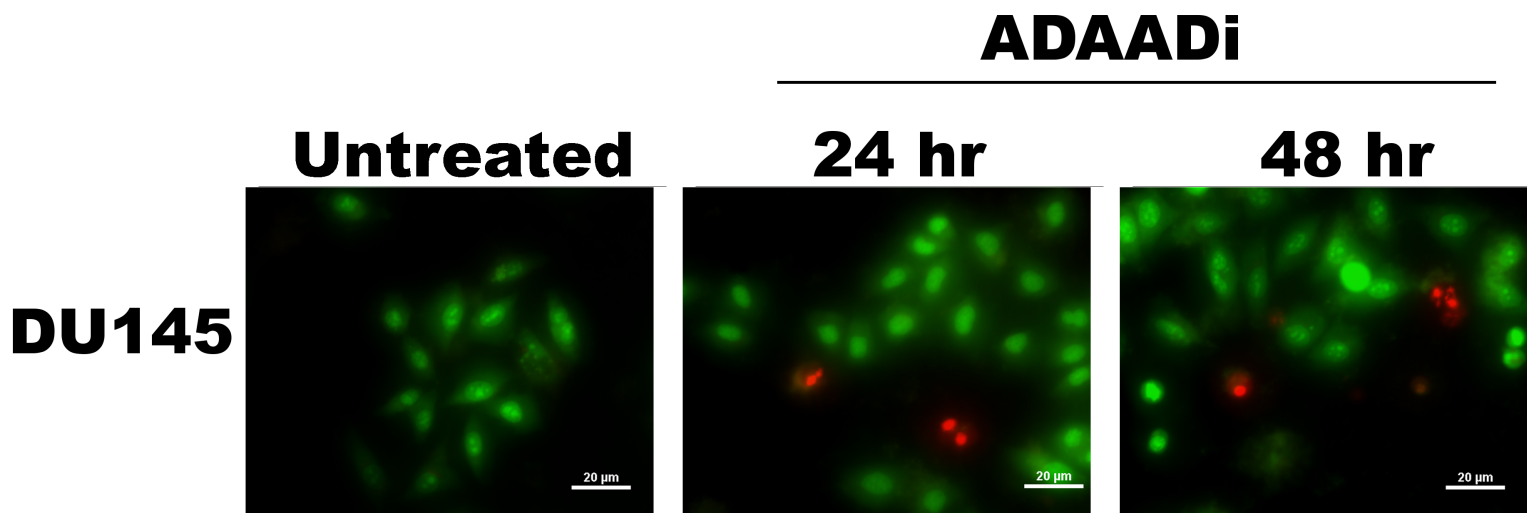

E.

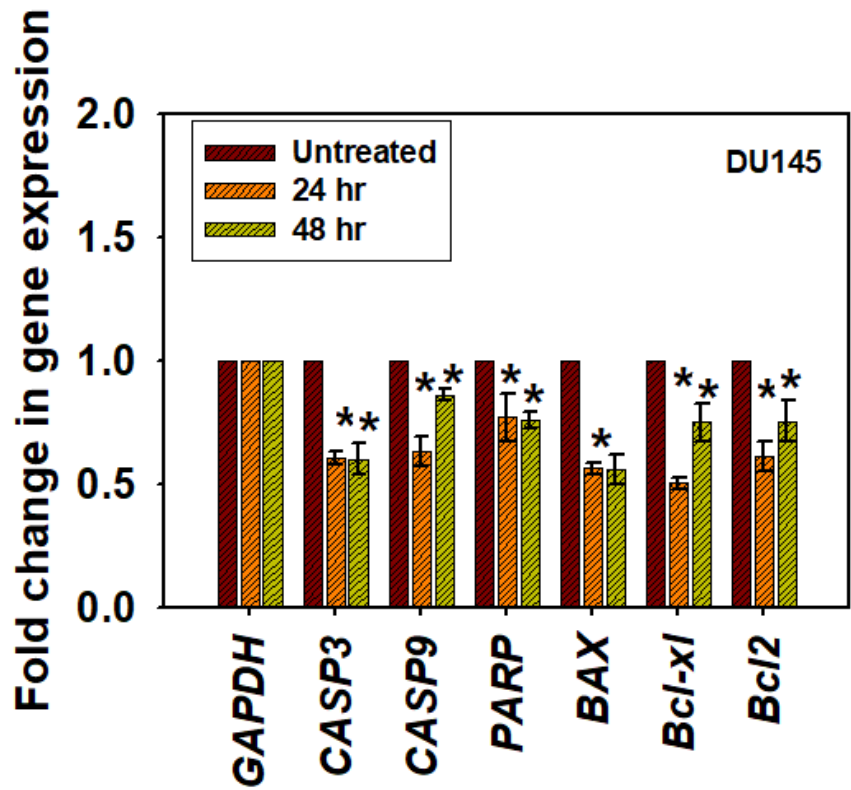

F.

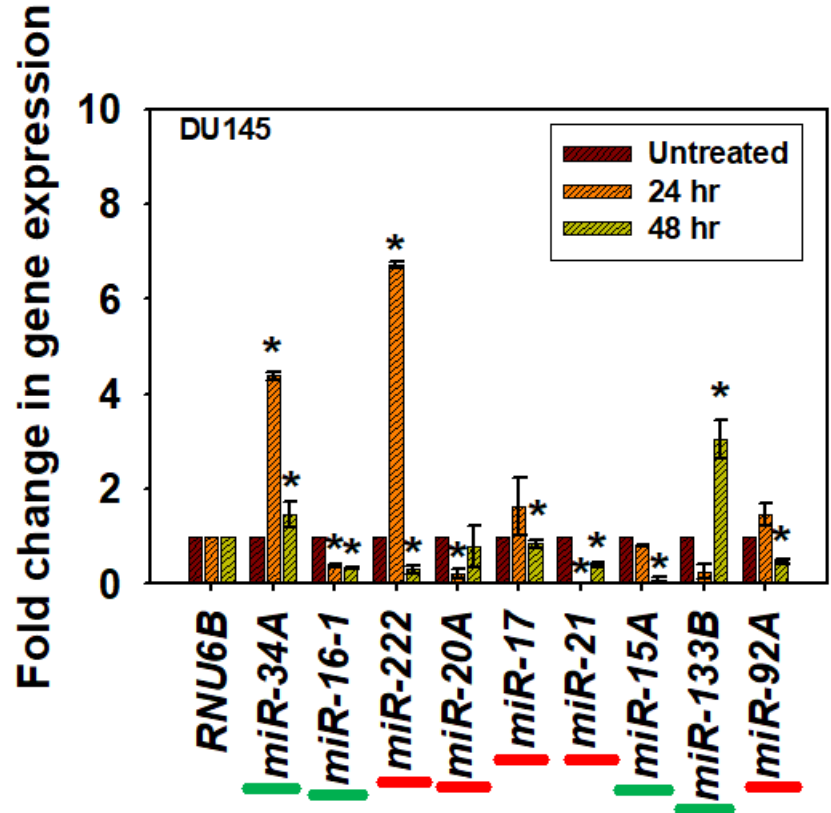

Supplement: S4 Fig — (A). A). FITC-Annexin V and PI staining in DU145 cells was monitored using FACS in untreated and ADAADi treated cells. (B). Quantitation of the live and apoptotic cells from FACS analysis for DU145 cells. The data is presented as average ± s.d. of three independent experiments. (C). Confocal images obtained after staining untreated and ADAADi treated DU145 cells with acridine orange and ethidium bromide. Green stained cells are viable cells while orange/red cells indicate non-viable or apoptotic cells. (D). The number of viable and apoptotic cells were counted in the untreated and treated DU145 cells. The results are presented as average ± s.d. of two independent experiments. In each experiment, more than 100 cells were counted. (E). (A). The transcript level of CASP3, CASP9, PARP, BAX, Bcl-xl and Bcl2 was measured using qPCR in DU145 cells after treatment with 2 μM ADAADi. GAPDH was used as the internal control in these experiments. (F). The levels of pro-apoptotic miRNA (underlined in green) and anti-apoptotic miRNA (underlined in red) were analyzed by qPCR in DU145 cells in absence and presence of 2 μM ADAADi. In these experiments, RNU6B was used as internal control. Statistical analysis for qPCR experiments was done using Sigma-plot and the star indicates significance at p<0.05. (PDF) [file pone.0251354.s004.pdf]

S5 Fig

A.

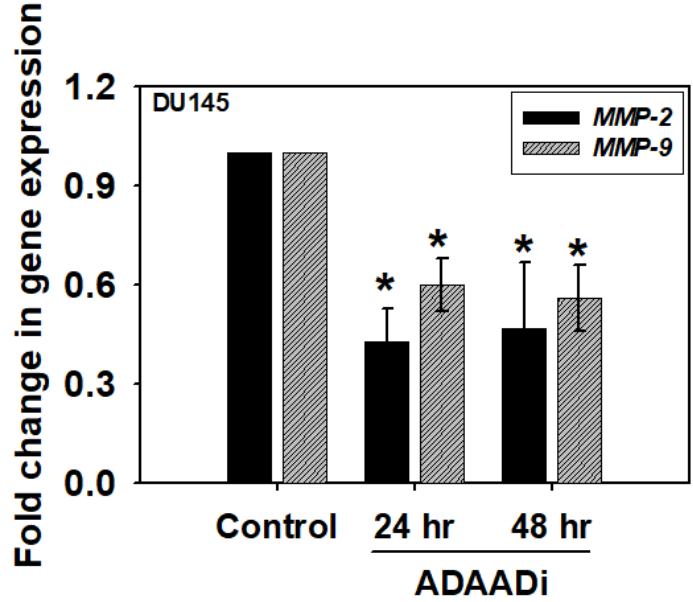

B.

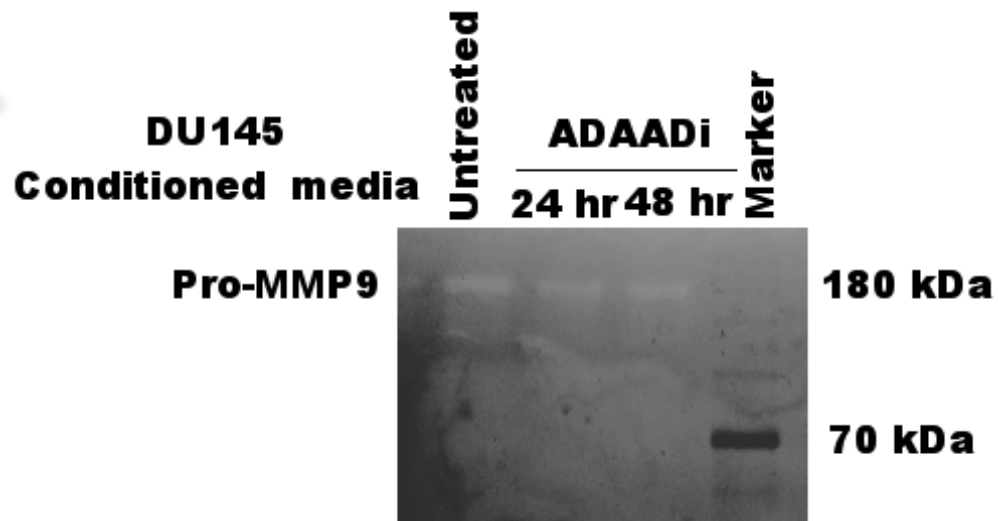

C.

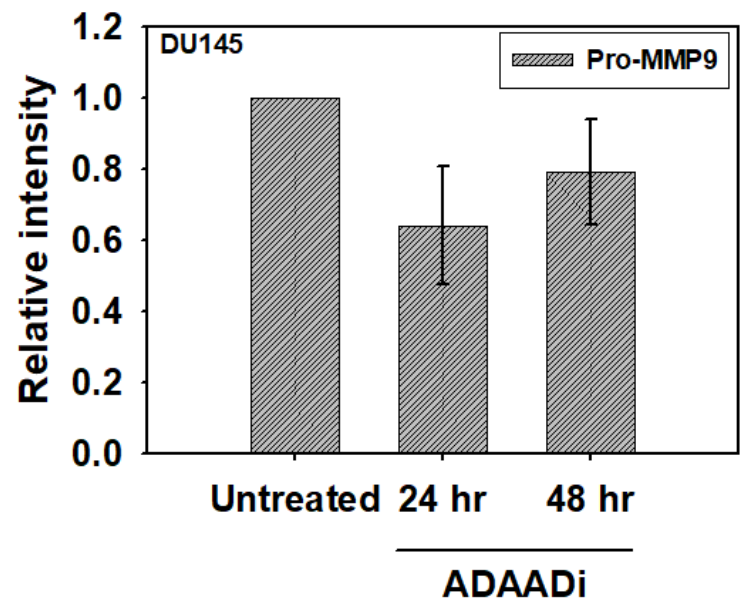

D.

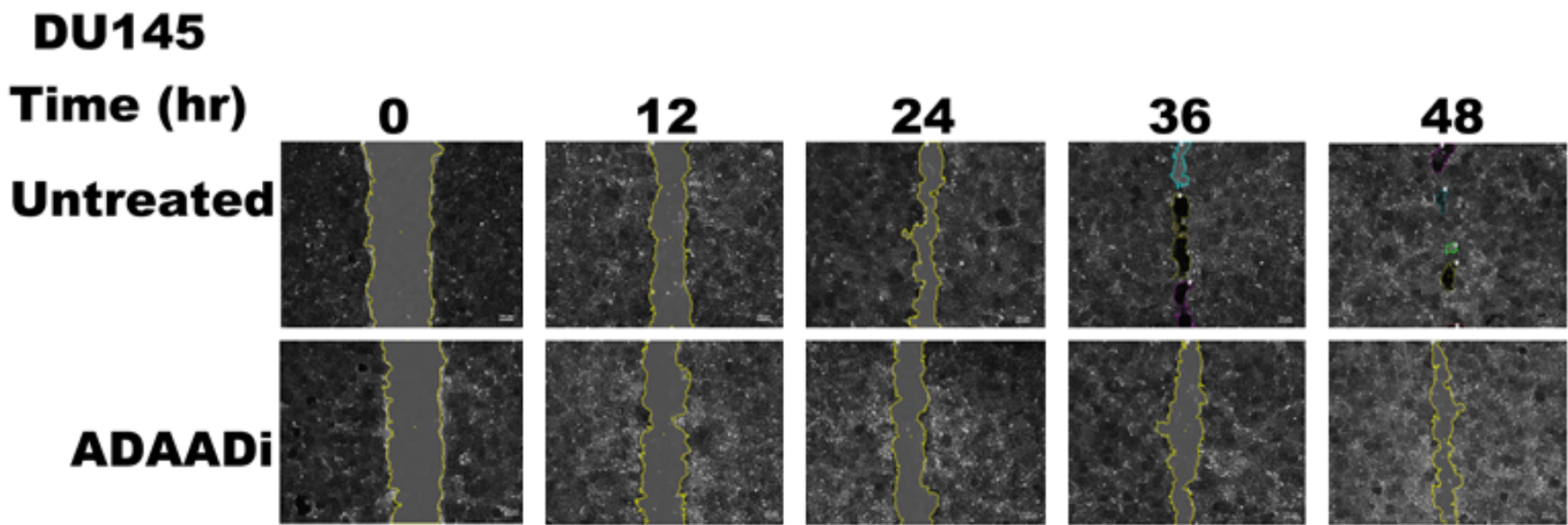

E.

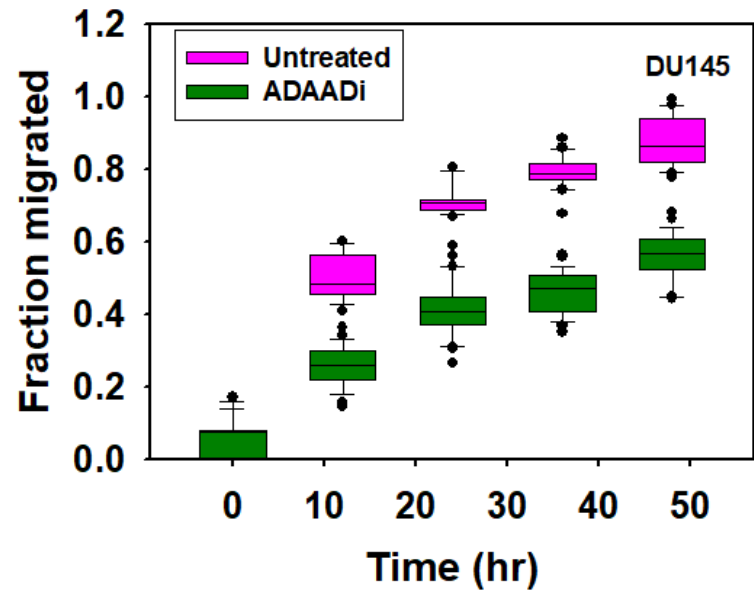

F.

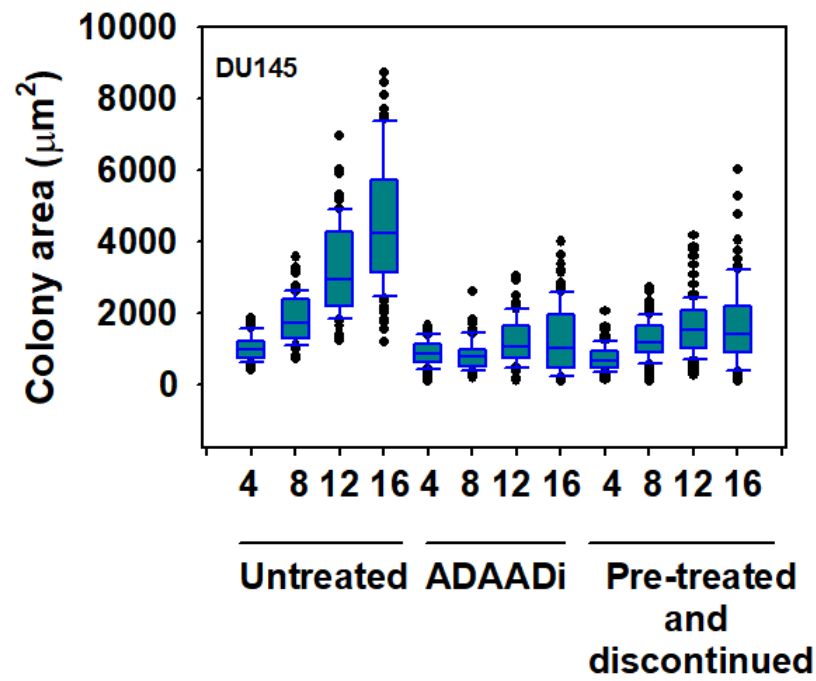

G.

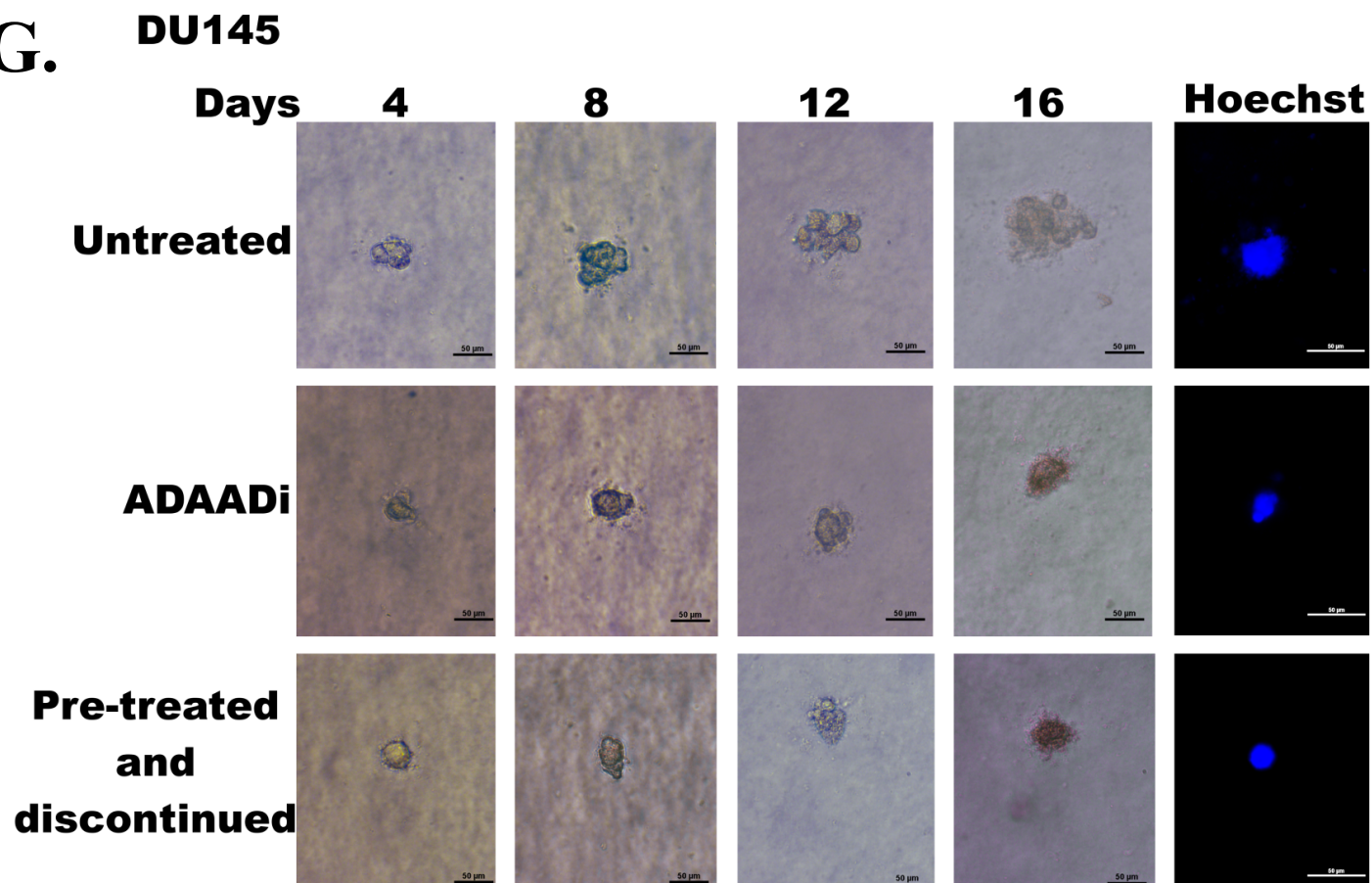

Supplement: S5 Fig — (A). qPCR analysis of MMP-2 and MMP-9 expression in untreated and ADAADi treated DU145 cells. (B). Zymography assay showing the secretion of Pro-MMP2 in the media by DU145 cells. (C). Quantitation of Pro-MMP2 was done using Image J software. The data was normalized with respect to untreated control and is presented as average ± s.d. of two independent experiments. (D). Image analysis of the wound assay captured at different time points after induction of gap in monolayer of DU145 cells. (E). Quantitation of the migration of DU145 cells in the absence and presence of ADAADi as a function of time. The data was normalized with respect to untreated control and is presented as average ± s.d. of three independent experiments. (F). Area (μm2) of the DU145 colonies calculated as a function of time in the absence and presence of ADAADi. (G). Colony formation monitored in untreated, ADAADi treated, and ADAADi pre-treated followed by discontinuation of the inhibitor in DU145 cells. On the 12th day, the colonies were fixed using 100% methanol and stained with Hoechst. Images were taken using Nikon TiS microscope. (PDF) [file pone.0251354.s005.pdf]

**S6 Fig**

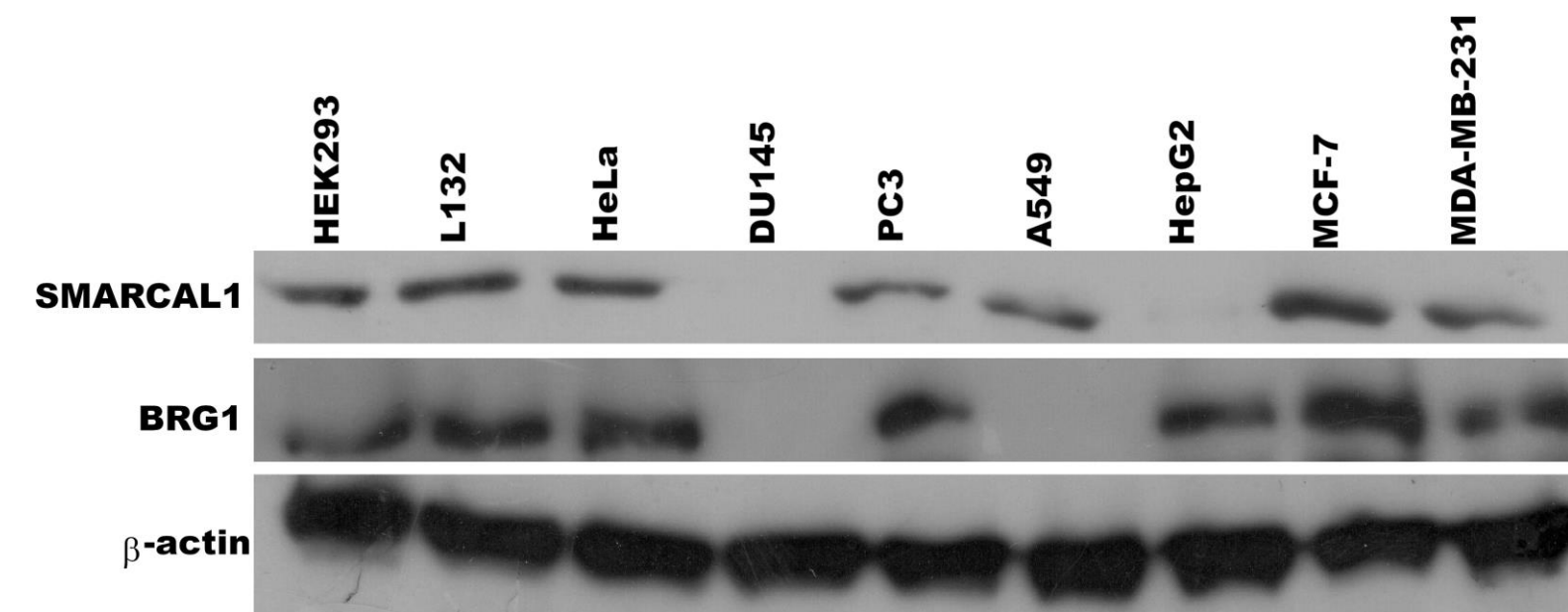

Supplement: S6 Fig — Expression of SMARCAL1 and BRG1 was analyzed by western blot in HEK293, L132, HeLa, DU145, PC3, A549, HepG2, MCF-7, and MDA-MB-231. β-actin was used as loading control. (PDF) [file pone.0251354.s006.pdf]

S7 Fig

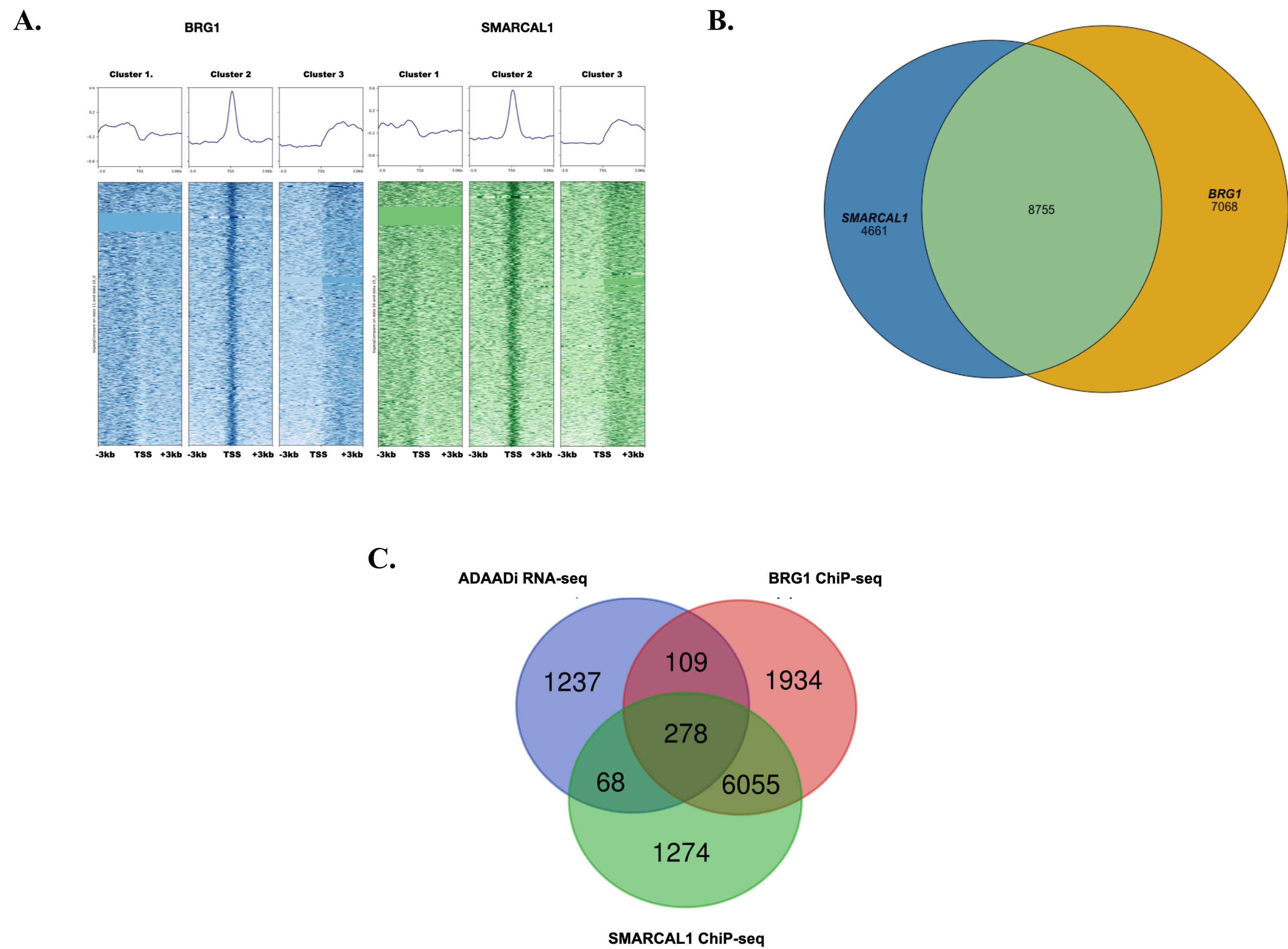

Supplement: S7 Fig — (A) Heat map of chip peaks (BRG1-blue, SMARCAL1-green) after Unsupervised k-mean (k = 5) clustering. SMARCAL1 and BRG1 shows peak occupancy over TSS on cluster 2. There was no binding over TSS on cluster 3 and 4. (B) ChIP-seq peak intersection between SMARCAL1 and BRG1. (C) Intersection among genes occupied by SMARCAL1, BRG1 and differentially expressed genes (padj<0.1) after ADAADi treatment (Fisher’s exact test, p = 1). (PDF) [file pone.0251354.s007.pdf]

S8 Fig

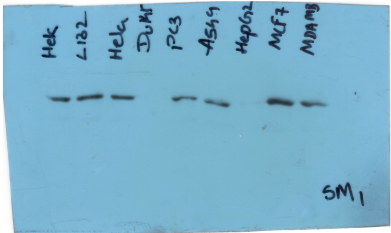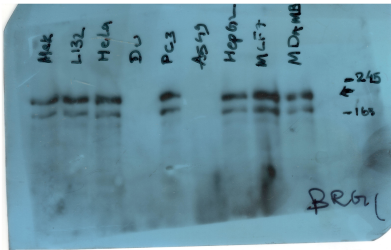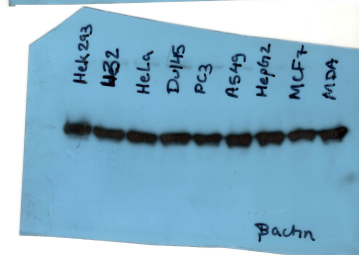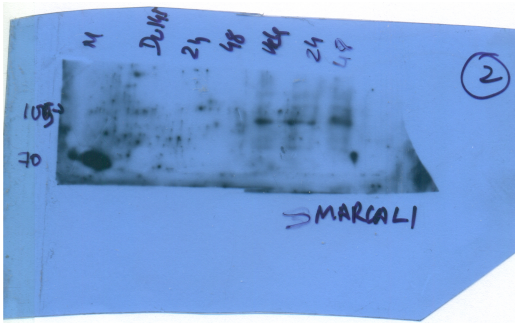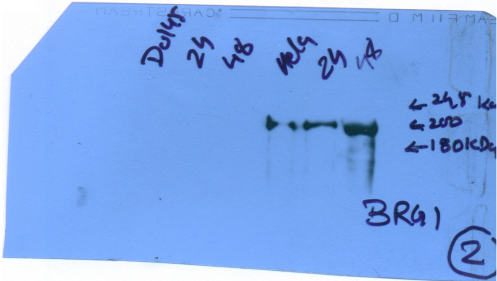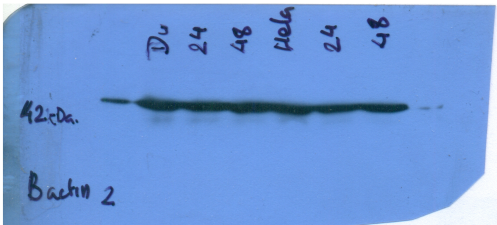

Marker Control 24 hr ADAADI 48 hr ADAADI

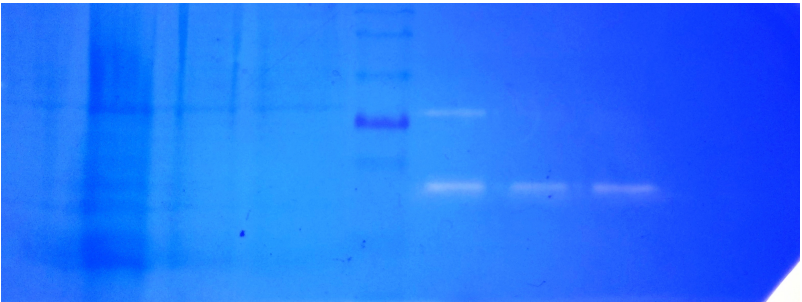

Control 24 hr ADAADI 48 hr ADAADI Marker

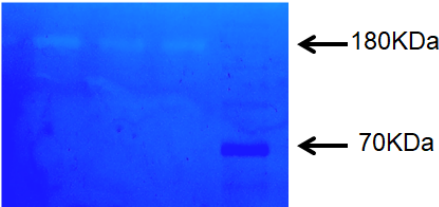

Supplement: S8 Fig — (PDF) [file pone.0251354.s008.pdf]
